# A New Dental Journal

**Published:** 1894-12

**Authors:** 


### EDITORIAL.

### A New Dental Journal.

There has just come to hand the Prospectus, of "The Dental Digest" a new Dental Journal, to be issued in the interests of the Dental Protective Association, and its ally, The Dental Protective Supply Company, both having headquarters in Chicago.

"The Dental Digest is not to be a rival of any of the journals now published, and will be unlike them in some important features."

The Digest will be under the control of Dr. J. N. Crouse, which is a sufficient guarantee of the propelling power behind and that will guide its destiny.

The Digest will be of interest to all the members of the Dental Protective Association.

